# Biological predictive factors in rectal cancer treated with preoperative radiotherapy or radiochemotherapy

**DOI:** 10.1038/sj.bjc.6604131

**Published:** 2007-12-18

**Authors:** F V Negri, N Campanini, R Camisa, F Pucci, S Bui, G Ceccon, R Martinelli, M Fumagalli, P L Losardo, P Crafa, C Bordi, S Cascinu, A Ardizzoni

**Affiliations:** 1 U.O. Oncologia Medica, Azienda Ospedaliero-Universitaria di Parma, via Gramsci, Parma 43100, Italy; 2 Istituto di Anatomia Patologica, Azienda Ospedaliero-Universitaria di Parma, Parma, Italy; 3 Servizio di Radioterapia, Azienda Ospedaliero-Universitaria di Parma, Parma, Italy; 4 Clinica di Oncologia, Azienda Ospedaliera Ospedali Riuniti-Università Politecnica delle Marche, via Conca, Ancona 60020, Italy

**Keywords:** biological predictive factors, rectal cancer, thymidylate synthase

## Abstract

We analysed the expression of microsatellite instability, p53, p21, vascular endothelial growth factor and thymidylate synthase (TS) in pretreatment biopsy specimens from 57 locally advanced rectal cancers. The aim of the study was to correlate the expression of these markers with pathological response. Nineteen patients were treated with preoperative concomitant radiotherapy (RT) and fluorouracil/oxaliplatin-based chemotherapy (RCT), while 38 had RT alone. Pathological complete remission (pCR) and microfoci residual tumour (micR) occurred more frequently in patients treated with RCT (*P*=0.002) and in N0 tumours (*P*=0.004). Among patients treated with RCT, high TS levels were associated with a higher response rate (pCR+micR; *P*=0.015). No such correlation was found in the RT group. The other molecular factors were of no predictive value. Multivariate analysis confirmed a significant interaction between nodal status and the probability of achieving a pathological response (*P*=0.023) and between TS expression and treatment, indicating that a high TS level is predictive of a higher pathological response in the RCT subset (*P*=0.007). This study shows that lymph node status is the most important predictive factor of tumour response to preoperative treatment. Thymidylate synthase expression assessed immunohistochemically from pretreatment tumour biopsies may be a useful predictive marker of rectal tumour response to preoperative RCT.

Preoperative radiation therapy alone (RT) or combined with chemotherapy (RCT) and improvements in surgical techniques, particularly the standardisation of total mesorectal excision (TME), have led to improved outcomes in the management of locally advanced rectal cancer in recent years (Swedish Rectal Cancer Trial, 1997; Sauer *et al*, 2004). With this approach, pathologic complete response (pCR), which is an important clinical predictor for both local control and disease-free survival, is achieved in 7–31% of patients (Ruo *et al*, 2002). In addition, obtaining a complete or near-complete pathologic response before surgery may increase the number of sphincter-sparing procedures (Ruo *et al*, 2002). The ability to predict tumour response before treatment may significantly impact the selection of patients for preoperative combined-modality therapy as well as potentially modify postoperative treatment plans.

Thymidylate synthase (TS), the target enzyme of the antimetabolite 5-FU, has been shown to be an independent prognostic marker of 5-FU chemotherapy in gastrointestinal tumours. Several preclinical and clinical studies have demonstrated that high TS levels correlate with 5-FU resistance in various malignancies (Leichman *et al*, 1997; Aschele *et al*, 1999). However, most of these studies dealt with outcome prediction for postoperative chemotherapy or treatment of metastatic disease rather than preoperative treatment. Therefore, the potential of TS expression levels to predict response to preoperative combined-modality therapy remains unsettled.

Little is known about other potential biological markers, such as p53, p21 and radio- and chemosensitivity of rectal cancer cells. Vascular endothelial growth factor (VEGF) is a potent mediator of tumour angiogenesis and has currently been assessed as a response predictor in rectal cancer. The results of a recently published study show that VEGF assessed immunohistochemically from pretreatment tumour biopsies may be a useful marker for rectal tumour response to preoperative RT (Zlobec *et al*, 2005a).

The aim of the present study was to test the predictive value of a number of tissue biomarkers, including TS, VEGF, p53, p21, MLH1 and MSH2 with regard to preoperative RT alone or RCT in rectal cancer.

## MATERIALS AND METHODS

A total of 57 patients with stage II and III rectal cancer consecutively treated at our Institution were included in this study, provided that adequate archive tumour tissue from pretreatment biopsy was available for biological studies. Disease staging was by computed tomography scan in all patients. Endorectal ultrasonography was performed in 17 patients. Thirty-eight patients were treated with preoperative RT alone by using high energy Linac (total dose 40 Gy specified to the isocentre, 250 cGy day^−1^, four fractions per week), delivered in 16 fractions, to include the true pelvis (rectal volume, perirectal, presacral and iliac nodes) with the three-field technique and shaped portals. Another 19 patients were treated within a phase II trial with preoperative radiation (total dose 45 Gy to the isocentre, 180 cGy day^−1^, with the three-field technique and shaped portals to cover the rectal volume and the perirectal, presacral and the internal iliac lymph nodes) and concurrent continuous infusion 5-FU at a daily dose of 200 mg m^−2^ and weekly oxaliplatin 60 mg m^−2^ for 5 weeks. The trial was approved by the Ethics Committee of our Institution. Informed consent was obtained from all patients. All the patients underwent surgery regardless of radiological response, which was not routinely performed. Standard surgery, including total mesorectal excision, was performed in all patients after an interval of approximately 6 weeks after completion of treatment. Further adjuvant treatment after surgery was left to the discretion of the treating physician. Only 18 of the 57 patients (32%) received in addition 5-FU-based adjuvant chemotherapy.

### Immunohistochemistry

Pretreatment tumour biopsies were collected from 57 patients. The specimens containing tumour were routinely fixed in buffered formalin and embedded in paraffin. Sections (4 *μ*m) were stained with haematoxylin and eosin for histological diagnosis and with the following primary antibodies: anti-HMSH2 (clone F11; Oncogene Research Products, Cambridge, MA, USA; working dilution 1/20); anti-HMLH1 (clone G168–728; Pharmingen, San Diego, CA, USA; working dilution 1/100); anti-p53 (clone DO-7; DakoCytomation, Glostrup, Denmark; working dilution 1/100); anti-p21waf (clone DCS-60.2; Neomarkers, Runcorn, UK; working dilution 1/20); anti-TS (clone TS 106; Chemicon; working dilution 1/50) and rabbit anti-VEGF (BioGenex, San Ramon, CA, USA; working dilution 1/50). For antigen retrieval, sections were treated with 10 mM citrate at pH 6.0 in a 750 W microwave oven for three 5-min cycles. The sections were immunostained with HRP Polymer (Ultravision LP Large Volume Detection System; Lab Vision) in accordance with the manufacturer's specifications. Diaminobenzidine was used for staining development and the sections were counterstained with haematoxylin. Negative controls consisted of substituting normal mouse serum for the primary antibodies.

#### Semiquantitative analysis

Immunostaining for HMSH2 and HMLH1 was estimated on a semiquantitative score according to the number of positive tumour cells as follows: 0% (0), <10% (1), 10–50% (2), 51–80% (3) or >80% (4). The intensity of staining was also evaluated as weak (1+), moderate (2+) or strong (3+). For each tumour case, the values for the two variables were multiplied, resulting in a score ranging from 0 to 12. The 0–6 scores were considered as altered expression and 7–12, as preserved expression. Vascular endothelial growth factor staining was considered positive in the tumour cell cytoplasm; immunoreactivity was graded as follows: positive, more than 10% of carcinoma cells stained, and negative less than 10% of carcinoma cells stained. For the evaluation of p53 and p21waf expression, immunostaining were classified into two groups, corresponding to the percentage of nuclear staining: negative expression (less than 10% positive tumour cells) and positive expression (more than 10% positive tumour cells). Thymidylate synthase expression was quantitated using a visual grading system based on the intensity of staining and was classified into groups from 0 to 3, where 0 and 1 were defined as low intensity, and 2 and 3 were defined as high intensity staining.

Assessment of immunoreactivity from pretreatment tumour biopsies was performed independently by two observers (PC and CB), blinded to postoperative tumour response. In case of disagreement, the relevant biopsies were re-examined simultaneously by both pathologists until an agreement was reached.

### Response classification

Tumour response was evaluated pathologically on postoperative specimens. Pathological complete remission was defined as no evidence of residual carcinoma or ypT0N0. Partial response was characterised by the presence of residual carcinoma microfoci (micR) typically measuring from 0.3 to 0.9 cm in diameter. Non-responsive tumours (NR) have large residual carcinoma. For the correlation between biomarkers and response to treatment, we separated the patients into two groups: responding (pCR, and partial response characterised by the presence of micR; pCR+micR) *vs* NR (Zlobec *et al*, 2005b).

### Statistics

Fisher's exact test was used to compare the distribution of the dichotomised molecular markers and tumours' characteristics in the two groups (responders and non-responders). *P*<0.05 was considered statistically significant. To assess the presence of a significant influence of treatment, tumour stage and molecular markers on response, the multivariate logistic regression model was applied, including in the model the appropriate interaction terms between treatment and TS status. The SPSS Software (version 8.0) was used in all analysis.

## RESULTS

Patient and tumour characteristics are summarised in Table 1. There were 32 men (56%) and 25 women (44%). Median age was 66 years (range 33–88). Thirty-eight patients (67%) were treated with RT only and 19 (33%) with RCT. An anterior resection was performed in 38 of 57 patients (67%). Only two patients treated with RT alone had an incomplete resection (R1). Nodal involvement was observed in 33 patients (58%). Eighteen percent of the patients (6/33 patients) with N1–2 tumours received RCT and 82% (27/33 patients) received RT alone.

Overall, pCR was observed in six patients (11%; 95% CI, 2.9–19.1%). In a further seven patients (12%; 95% CI, 3.6–20.4%), only microscopic tumour foci were found. Pathologic tumour response by treatment is shown in Table 2. Among patients treated with RCT, pCR was observed in four patients (21%; 95% CI, 2.7–39.3%) and another five patients (26%; 95% CI, 6.3–45.7%) had only micR (pCR+micR=47%). In the RT group, pCR was observed in two patients (5%; 95% CI, −1.9–11.9%) and another two patients (5%; 95% CI, −1.9–11.9%) had only micR (pCR+micR=10%) (*P*=0.002; Table 2).

Table 2 shows response rates according to various clinical and biological parameters. Forty-seven percent of the patients (9/19 patients) with N0 tumours experienced an objective response. In N1–2 tumours, only 9% of the patients (3/33 patients) responded to treatment (*P*=0.004; Table 2). In the present series, overall all the other variables, including T status, TS, VEGF, p53, p21, MLH1 and MSH2 expression, failed to affect the probability of response (Table 2).

Table 3 shows the subgroup exploratory analysis by treatment according to TS status. Interestingly, among patients treated with RCT, the percentage of pCR+micR was significantly greater in tumours with high TS expression levels than in those with low TS levels (88 *vs* 12%; *P*=0.015). In contrast, the response rate in RT group was not affected by TS expression (13 *vs* 9%, respectively). Subgroup analysis confirmed the lack of predictive value of p53, p21, VEGF, MLH1 and MSH2.

To assess the effect of treatment and molecular markers on response, a regression logistic model was used, with the probability of achieving a response as dependent variable and TS status, nodal status, treatment and the interaction terms as covariates. Besides nodal status (odds ratio (OR)=8.0; 95% CI 1.34–47.8, *P*=0.023), only the interaction of TS and treatment was kept in the model as independent predictive factors for response (OR=30.6; 95% CI 2.5–371.7, *P*=0.007). As a consequence of this interaction, the increased activity seen in tumours expressing high TS levels in patients treated with preoperative RCT was not seen in patients treated with RT only (OR=1.5; 95% CI 0.14–16.3).

## DISCUSSION

Multidisciplinary treatment of rectal cancer has evolved over the years from adjuvant chemoradiation to preoperative radiation and, more recently, to preoperative combined chemoradiation, in view of an improved toxicity and local control (Sauer *et al*, 2004).

Oxaliplatin added to FU was found to enhance the efficacy in both metastatic and adjuvant colon cancer setting and may act as a potent radiation sensitiser. For these reasons, this regimen is also being used increasingly in preoperative treatment of rectal cancer. Concomitant RT and oxaliplatin with either FU/LV or capecitabine can achieve pCR rates in 15–28% in locally advanced rectal cancer (Aschele *et al*, 2005; Chau *et al*, 2006). The results of the present study show that, besides disease extent, the type of treatment is the most important predictor of tumour response, with patients treated with combined RCT having a higher probability of achieving a pCR compared to those treated with RT alone (*P*=0.002). A pCR rate of 21% with an additional 26% micR rate in the RCT group in this study is encouraging. A high response rate is of clinical significance in rectal cancer because patients who achieve a complete or near-complete pathologic response may experience improved long-term local control and overall survival (Ruo *et al*, 2002). Therefore, the identification of distinct clinical, pathological and molecular parameters, with the ability to predict response to preoperative RCT, may become a useful tool in the therapeutic management of rectal cancer.

Thymidylate synthase plays an important role in pyrimidine nucleotide synthesis and represents an important chemotherapeutic target for 5-FU. Overall, the proportion of colorectal cancers expressing high levels of TS is about 50% (range, 14–80%) in both advanced and adjuvant settings (Edler *et al*, 2002; Johnston *et al*, 2003). In our study, the percentage of low and high TS expressors was 39 and 56%, respectively.

Several studies have been published during the past few years relating to the measurement of expression of TS to response to fluoropyrimidines as well as to the overall outcome of patients with colorectal carcinoma. A recent meta-analysis showed that colorectal cancers expressing TS at high levels seem to be associated with a poorer prognosis compared with low TS-expressing tumours, although in the adjuvant setting this seems to be the case only for patients treated by surgery alone. In patients treated with both surgery and adjuvant 5-FU, TS expression does not seem to predict clinical outcome (Popat *et al*, 2004).

A direct correlation between TS expression and response to 5-FU has been reported both *in vitro* and *in vivo*. Preclinical studies have demonstrated that an increase in TS expression is associated with resistance to fluoropyrimidines (Johnston *et al*, 1992). In advanced colorectal cancer, patients with high levels of TS are unlikely to respond to 5-FU, whereas patients with low levels have higher than expected response rates (>50%) (Aschele *et al*, 1999; Cascinu *et al*, 1999).

To date, only a small number of studies have concentrated on the role of TS and tumour response in rectal cancer patients, especially 5-FU-based chemoradiotherapy. In rectal cancer, low TS gene expression has been found to correlate significantly with tumour response after neoadjuvant 5-FU-based CRT (Jakob *et al*, 2005). In contrast with these results, the present study indicates that tumours responding to preoperative RCT most often express high levels of TS in their pretreatment biopsies, whereas non-responding tumours are generally poorly immunoreactive.

Several factors can account for the controversial results on the predictive/prognostic role of TS expression. The first is related to the different techniques used to assess TS expression. The commonest technique used is IHC (Popat *et al*, 2004). Several semiquantitative methods for dichotomising TS expression have been used. The most common method relates TS expression to chromagen intensity with either a 4- or 5-grade scale. Some studies further subcategorised staining by a either focal or diffuse pattern. In a recently published study, for example, a significant correlation between protein expression and tumour response in rectal cancer patients was seen only when both staining intensity and staining pattern were considered, with a significant association between high TS expression in tumour biopsies and non-response to therapy (*P*=0.04) (Jakob *et al*, 2005). In our study, we used a four chromagen intensity grade, ranging from 0 to 3, where 0 and 1 were defined as low intensity, and 2 and 3 were defined as high intensity staining, as reported previously (Allegra *et al*, 2002). The existence of such a high variability supports the demand for a more uniform evaluation of IHC expression.

Another reason for the contradictory results may be related to the different FU schedule used. FU may act as two different drugs according to the mode of administration. Bolus FU may exert its major effect on RNA, whereas continuous infusion may have a preferential effect on TS (Sobrero *et al*, 1997). Most published studies of patients with advanced colorectal cancer indicate that patients with high tumour levels of TS are unlikely to respond to infusional treatment with 5FU (Leichman *et al*, 1997; Aschele *et al*, 1999). In contrast, in the present study, FU was administered as continuous infusion at a daily dose of 200 mg m^−2^ and high TS levels were found to be predictive of response to RCT.

There is some evidence that high TS protein expression may predict high sensitivity to FU in the adjuvant setting. At least four studies have suggested that patients whose primary tumours had high TS expression may benefit from FU-based adjuvant treatment, while those whose tumours had low expression do not (Johnston *et al*, 1994; Yamachika *et al*, 1998; Takenoue *et al*, 2000; Edler *et al*, 2002). In rectal cancer, adjuvant FU-based chemotherapy demonstrated significant improvement in disease-free and overall survival for rectal cancer patients with high TS levels (Johnston *et al*, 1994). To our knowledge, this is the first study to suggest that patients who have high TS-expressing rectal cancer have a higher probability to respond to preoperative CRT.

It should also be noted that many more studies were conducted regarding the prognostic value of TS expression in primary colorectal cancer treated with postoperative chemotherapy and the role of TS expression as a predictor of chemotherapeutic benefit in metastatic disease rather than response prediction to preoperative therapy. Importantly, TS expression measured in primary tumours may not reflect TS levels in lymph node or other metastases (Marsh *et al*, 2002).

Moreover, evidence has accumulated that oxaliplatin treatment results in downregulation of TS expression, providing a possible explanation, although still speculative, for the higher response rate of the combination of oxaliplatin and FU, compared with oxaliplatin alone, in FU-resistant patients (Yeh *et al*, 2004). In addition, it has been shown that oxaliplatin remains highly cytotoxic in cells that overexpress TS. In breast cancer-derived cell line with tetracycline-regulated expression of TS, TS overexpression confers an increased resistance to TS-targeted drugs, while the growth inhibitory effect of other drugs such as oxaliplatin is unaffected by TS upregulation (Longley *et al*, 2001). The use of an oxaliplatin-containing chemotherapy in our study could explain our findings. Furthermore, the absence of TS effect on RT treatment makes our hypothesis sound.

When we explored the potential predictive value of other molecular markers, we found no effect on response. Contrary to a previous study on rectal cancer patients receiving preoperative RT, showing that VEGF expression in NR was significantly greater than in completely responsive tumours, the present study does not demonstrate a significant role of VEGF overexpression on clinical response (Zlobec *et al*, 2005a). Also, the present data do not confirm the previously reported value of high microsatellite instability, p21WAF1/C1PI and p53 for predicting tumour response to preoperative RT or RCT (Charara *et al*, 2004; Kelley *et al*, 2005; Komuro *et al*, 2005). These conflicting results may be attributable to the retrospective nature and the limited detection power inherent in studies that test small subsets of patients.

An interesting finding, although not directly related to the goal of this work, was the observation that lymph node status was significantly associated with tumour response. Lymph node status was first demonstrated to have prognostic value in patients with locally advanced rectal cancer treated on the CAO/ARO/AIO-94 trial of the German Rectal Cancer Study Group (Liersch *et al*, 2006). All patients who developed cancer recurrence had a persistently positive lymph node status after therapy, which reflects the poor prognosis of these patients. In our study, lymph node status turned out to be the most important predictive factor, with a significantly higher response rate in N0 tumours as compared with N1–2 tumours (47 *vs* 9%; *P*=0.004), independently of treatment and TS status.

In conclusion, despite the small sample size and the retrospective nature of the study, our data indicate that lymph node status is the most important predictive marker of tumour response to preoperative treatment in rectal cancer. In addition, among a variety of biological markers evaluated, immunohistochemical assessment of TS from pretreatment tumour biopsies was the only one with potential for the prediction of tumour response to preoperative oxaliplatin-based RCT. This observation may deserve further validation in prospective studies of FOLFOX-RT regimens.

## Figures and Tables

**Table 1:** Baseline characteristics

**Baseline characteristic**	**Number of patients (*N*=57)**	**%**
*Age, years*		
Median	66	
Range	33–88	
		
*Sex*		
Male	32	56
Female	25	44
		
*Tumour stage*		
T1–2	12	21
T3–4	43	75
N0	19	33
N1–2	33	58
		
*Preoperative treatment*		
RT	38	67
RCT	19	33
		
*Surgery*		
Abdominoperineal resection	15	26
Sphincter-preserving surgery	38	67
		
*TS*		
Low	22	39
High	32	56
		
*P53*		
Negative	13	23
Positive	44	77
		
*VEGF*		
Negative	21	37
Positive	31	54
		
*P21*		
Negative	24	42
Positive	32	56
		
*MLH1*		
Preserved	43	75
Altered	14	25
		
*MSH2*		
Preserved	44	77
Altered	13	23

Abbreviations: RT=radiotherapy; RCT=radiochemotherapy; TS=thymidilate synthase; VEGF=vascular endothelial growth factor.

**Table 2:** Response according to various clinical and pathological parameters

**Parameter**	**Response**	**%**	**No response**	**%**	***P*-value**
*Tumour stage*					0.71
T1–2	2	17	10	83	
T3–4	11	26	31	74	
					
*Nodal status*					0.004
N0	9	47	10	53	
N1–2	3	9	30	91	
					
*Treatment*					0.002
RT	4	10	34	90	
RCT	9	47	10	53	
					
*TS*					0.20
Low	3	14	18	86	
High	10	32	21	68	
					
*P53*					0.71
Negative	2	15	11	85	
Positive	11	26	31	74	
					
*VEGF*					0.31
Negative	7	33	14	67	
Positive	5	17	24	83	
					
*P21*					0.74
Negative	4	18	18	82	
Positive	8	25	24	75	
					
*MLH1*					0.15
Preserved	12	29	29	71	
Altered	1	7	13	93	
					
*MSH2*					0.71
Preserved	11	26	31	74	
Altered	2	15	11	85	

**Table 3:** Response to preoperative treatment according to TS level

**Treatment**	**TS level**	**Response**	**No response**	***P*-value**
CRT				
	High (*n*=8)	7 (88%)	1 (12%)	0.015
	Low (*n*=10)	2 (20%)	8 (80%)	
RT				
	High (*n*=23)	3 (13%)	20 (87%)	NS
	Low (*n*=11)	1 (9%)	10 (90%)	
